# Increased linear bone growth by GH in the absence of SOCS2 is independent of IGF‐1

**DOI:** 10.1002/jcp.25006

**Published:** 2015-07-27

**Authors:** Ross Dobie, Syed F. Ahmed, Katherine A. Staines, Chloe Pass, Seema Jasim, Vicky E. MacRae, Colin Farquharson

**Affiliations:** ^1^ The Roslin Institute and R(D)SVSUniversity of EdinburghEaster BushMidlothianUK; ^2^ Developmental Endocrinology Research Group, School of MedicineUniversity of GlasgowYorkhillGlasgow, ScotlandUK

## Abstract

Growth hormone (GH) signaling is essential for postnatal linear bone growth, but the relative importance of GHs actions on the liver and/or growth plate cartilage remains unclear. The importance of liver derived insulin like‐growth factor‐1 (IGF‐1) for endochondral growth has recently been challenged. Here, we investigate linear growth in Suppressor of Cytokine Signaling‐2 (SOCS2) knockout mice, which have enhanced growth despite normal systemic GH/IGF‐1 levels. Wild‐type embryonic ex vivo metatarsals failed to exhibit increased linear growth in response to GH, but displayed increased *Socs2* transcript levels (*P* < 0.01). In the absence of SOCS2, GH treatment enhanced metatarsal linear growth over a 12 day period. Despite this increase, IGF‐1 transcript and protein levels were not increased in response to GH. In accordance with these data, IGF‐1 levels were unchanged in GH‐challenged postnatal *Socs2^‐/‐^* conditioned medium despite metatarsals showing enhanced linear growth. Growth‐plate *Igf1* mRNA levels were not elevated in juvenile *Socs2^‐/‐^* mice. GH did however elevate IGF‐binding protein 3 levels in conditioned medium from GH challenged metatarsals and this was more apparent in *Socs2^‐/‐^* metatarsals. GH did not enhance the growth of *Socs2^‐/‐^* metatarsals when the IGF receptor was inhibited, suggesting that IGF receptor mediated mechanisms are required. IGF‐2 may be responsible as IGF‐2 promoted metatarsal growth and *Igf2* expression was elevated in *Socs2^‐/‐^* (but not WT) metatarsals in response to GH. These studies emphasise the critical importance of SOCS2 in regulating GHs ability to promote bone growth. Also, GH appears to act directly on the metatarsals of Socs2^‐/‐^ mice, promoting growth via a mechanism that is independent of IGF‐1. J. Cell. Physiol. 9999: 2796–2806, 2015. © 2015 Wiley Periodicals, Inc.

The anabolic role of growth hormone (GH) in long bones is well accepted. The relative contributions of GH acting on the liver (increasing growth promoting endocrine factors) or directly (on growth plate cartilage) however remain unclear (Lupu et al., 2001). It is likely that both these modes of GH action function in a highly coordinated manner to regulate growth plate function and linear bone growth. GH increases insulin like growth factor‐1 (IGF‐1) production in a number of tissues. Specifically, GH acting on the liver results in an increase in circulating IGF‐1 which functions in an endocrine manner (Sjogren et al., 1999; Yakar et al., 1999). GH induced IGF‐1 production within the growth plate cartilage functions in an autocrine/paracrine manner. Furthermore, GH may also act on the growth plate via IGF‐1 independent mechanisms (Gevers et al., 2002a; Wang et al., 2004).

As expected, studies with Snell (*dw/dw*) and Ames (*df/df*) hypopituitary dwarf mice reveal a reduction in body weight and growth retardation. Mice with global inactivated GHR (*ghr*), GH‐ releasing hormone receptor (*ghrhr*), IGF‐1 (*Igf1*), IGF‐1 receptor (IGF‐1R; *Igf1r*), and insulin receptor substrate‐1 (*Irs‐1*) have a similar phenotype (Sinha et al., 1975; Smeets and Vanbuuloffers, 1983,1983; Li et al., 1990; Sornson et al., 1996; Wang et al., 2004).

The importance of circulating IGF‐1 on linear growth was challenged by two independent studies. Liver‐specific IGF‐1 deficient (LID) mice have a significant reduction of circulating IGF‐1, but body weight and bone length were essentially normal (Sjogren et al., 1999; Yakar et al., 1999). Furthermore, a phenotypic comparison of LID, acid labile subunit knockout (ALSKO), insulin like growth factor binding protein (IGFBP)‐3 knockout (BP3KO), and triply deficient LID/ALSKO/BP3KO mice indicated that while all had decreased serum IGF‐1 levels this did not predict linear growth potential (Yakar et al., 2009). Compared to WT mice, ALSKO mice (60% reduction in serum IGF‐1) were 8% shorter, whereas BP3KO mice (40% reduction in serum IGF‐1) were 5% longer, and LID mice (80% reduction in serum IGF‐1) were of equal length. Most strikingly, despite virtually undetectable circulating IGF‐1 (2.5% of WT IGF‐1 levels), the triply deficient LID/ALSKO/BP3KO mice exhibited only a modest 6% decrease in body length, comparable to that of the ALSKO mice that had much greater serum IGF‐1 levels (Yakar et al., 2009). However, interpretation of some of these observations is complicated by a marked increase in circulating GH to supraphysiological levels (Yakar et al., 2002) and models with a physiological level of circulating GH maybe more informative.

Some function has been attributed to circulating IGF‐1, and it is possible that a threshold concentration of circulating IGF‐1 is required for normal linear growth (Lupu et al., 2001). The data from the LID/ALSKO/BP3KO mice however are at odds with this concept (Yakar et al., 2002; Yakar et al., 2009). When hepatic IGF‐1 production was achieved in mice lacking *Igf1* gene expression in all other tissues, it was found that circulating IGF‐1 contributes to 30% of the adult body size and sustains postnatal development (Stratikopoulos et al., 2008). Similarly, in *Igf1* null mice with hepatic over expression of the rat *Igf1, s*erum IGF‐1 production supported normal body growth during and after puberty, despite absence of tissue IGF‐1 (Wu et al., 2009; Elis et al., 2010).

Alternative strategies to delineate between circulating and local IGF‐1 effects on linear bone growth have involved the targeted deletion of *Igf1* in epiphyseal chondrocytes. These mice had a 40% reduction in cartilage IGF‐1 expression and normal circulating IGF‐1 levels (Govoni et al., 2007). Linear growth was reduced by 27% between 2 and 4 weeks of age, highlighting that local chondrocyte‐produced IGF‐1 is an important regulator of longitudinal growth. While highly informative, these studies fail to address the possibility of a GH action on the growth plate, which is independent of IGF‐1. Double *ghr/igf1* KO mice have a more severe phenotype than *ghr* or *igf1* KO's alone suggesting that there are GH actions on linear bone growth that are independent of IGF‐1 (Lupu et al., 2001).

A role for GH acting directly on growth plate cartilage is also suggested by data from suppressor of cytokine signaling 2 (SOCS2) null mice (Metcalf et al., 2000; MacRae et al., 2009). SOCS2 is expressed by epiphyseal chondrocytes, and is a recognised negative regulator of GH signaling via inhibition of the Janus kinase/signal transducers and activators of transcription (JAK/STAT) pathway (Hilton, 1999; Greenhalgh et al., 2002; Rico‐Bautista et al., 2006; Flores‐Morales et al., 2006; Pass et al., 2009). Intriguingly, these mice are characterized by increased growth, including increased long bone length and mass, without elevated circulating levels of IGF‐1 or GH (Metcalf et al., 2000; Greenhalgh et al., 2005; MacRae et al., 2009; Dobie et al., 2014). It can therefore be assumed that GH actions directly on growth plate cartilage (IGF‐1 dependent or independent) are driving this increased linear bone growth.

The ex vivo metatarsal culture method has been exploited in many studies as a method for analysing endochondral bone growth. This model provides a more physiological environment than cultured chondrocytes as chondrocyte interactions with each other and the extracellular matrix (ECM) are maintained. (Mushtaq et al., 2004; MacRae et al 2007; Chagin et al 2010). Studies have shown that the growth rate of embryonic bones in culture is similar to that found in vivo (Scheven & Hamilton 1991; Coxam et al., 1996). Furthermore, the ability to grow metatarsals in long‐term cultures without fetal bovine serum allows for the manipulation of medium conditions and investigation of the effects of various treatments. Recently, it has been reported that GH is able to stimulate longitudinal growth of *Socs2^‐/‐^* metatarsals, but not WT bones (Pass et al., 2012). The *Socs2^‐/‐^* metatarsal culture model is therefore a valuable model in investigating the mechanisms by which local GH enhances linear bone growth.

This study aimed to utilize the metatarsal organ culture model to explore the mechanisms by which linear bone growth is enhanced in *Socs2^‐/‐^* mice, and in doing so, establish the mechanisms of GH's control of growth plate function.

## Materials and Methods

### Mice

*Socs2^‐/‐^* mice were generated as previously described (MacRae et al., 2009). For genotyping, tail or ear biopsied DNA was analyzed by PCR for SOCS2 (WT) or the neocassette (*Socs2^‐/‐^*) Primer sequences can be found in Supp. Table S1 (Eurofins MWG Operon, London, UK) (Pass et al., 2012). All animal experiments were approved by Roslin Institute's Animal Users Committee, and the animals were maintained in accordance with Home Office (UK) guidelines for the care and use of laboratory animals.

### Growth analysis and growth plate dynamics

Six‐week‐old male WT and *Socs2* ^‐/‐^ mice received a subcutaneous injection of 10 mg/kg calcein (Sigma, Poole, UK) in sodium bicarbonate solution 2 days prior to sacrifice. Tibiae, fixed overnight in 4% paraformaldehyde (PFA) were embedded in methylmethacrylate and sections (5 μm) were cut and processed using standard procedures (Idris et al., 2009). The longitudinal bone growth rate was measured as previously described (Owen et al 2009; Pass et al., 2012). In brief, the distance between the original growth plate mineralization front and the final fluorescing mineralization front within the metaphysis was measured at 10 different points along the width of the section using image analysis software and a Leica DMBR fluorescent microscope. Measurements were divided by the number of days between injection and sacrifice (2 days) to give a bone formation rate per day. Four sections per tibia were measured from 6 mice per group.

### Embryonic and postnatal metatarsal organ culture

The middle three metatarsals were isolated from 17‐day‐old WT and *Socs2^‐/‐^* embryos (E17) or 3‐day‐old (PN3) WT and *Socs2^‐/‐^* pups. At both developmental ages the growth plate contains both proliferating and hypertrophic chondrocytes but the primary ossification center, while newly formed in E17 metatarsals, is almost completely developed in PN3 bones (van Loon et al 1995; Reno et al., 2006). Each bone was cultured individually in 1 well of a 24‐well plate (Costar, High Wycombe, UK) in 300 μl αMEM medium (without ribonucleosides) or 300 μl DMEM + F12 medium supplemented with 0.2% BSA (Fraction V), 5 μg/ml L‐ascorbic acid phosphate, 1 mM β‐glycerophosphate, 0.05 mg/ml gentamicin, 1.25 μg/ml fungizone (Invitrogen, Paisley, UK) as previously described (Chagin et al., 2010; Pass et al., 2012). The perichondrium and the primary ossification center was not removed prior to explant culture. Recombinant human (rh)GH and rhIGF‐1 (Bachem, Merseyside, UK; both 100 ng/ml) were added as used previously (Mushtaq et al., 2004a; MacRae et al., 2006a; Pass et al., 2012). Recombinant mouse (rm)IGF‐2 (R&D systems, Minneapolis, MN) was added at the same concentration as rhIGF‐1 (100 ng/ml). One micrometer NVP‐AEW541 (IGF‐1 receptor kinase inhibitor) (Garcia‐Echeverria et al., 2004; Gan et al., 2010) was added 16 h prior to the addition of GH. Bones were incubated in a humidified atmosphere (37°C, 5% CO_2_), for up to 13 days. Bone lengths were measured from articular surface to articular surface through the middle of the metatarsals using a Nikon eclipse TE300 microscope with a digital camera attached, using Image Tool (Image Tool Version 3.00, San Antonio, TX). For RNA extraction 3–4 bones were pooled in 100 µl Trizol reagent (Invitrogen) at days 7 and 12 of culture and RNA extracted according to the manufacturer's instructions (RNeasy Mini Kit, Qiagen). Conditioned medium was collected at days 5, 7, and 12 and stored at −80°C.

### Growth plate micro dissection and RNA extraction

Tibiae were dissected from 7‐week‐old male WT (n = 4) and *Socs2^‐/‐^* mice (n = 3). Bones were briefly immersed in 4% aqueous (wt./vol.) polyvinylalcohol (PVA; Grade GO4/140, Wacker Chemicals, Walton‐on‐Thames, UK), chilled by precipitate immersion in n‐hexane (BDH, Poole, UK; grade low in aromatic hydrocarbons) and stored at −80^°^C (25;26). Using optimal cutting temperature (OCT) embedding medium (Brights, Huntingdon, UK), 30 μm thick longitudinal sections of the proximal tibia were cut at −30°C (Brights, OT model cryostat), mounted on Superfrost Plus slides (Fischer Scientific, Chicago, IL) before storage at −80°C. Slides were briefly thawed as described previously (Nilsson et al., 2007; Staines et al., 2012), and then dehydrated in graded solutions of ethanol (70%, 95%, and 100%) with sections kept under a xylene droplet throughout the microdissection. The entire growth plate was dissected free from the perichondrium, the secondary ossification zone and the primary spongiosa. Growth plates from both tibae of each mouse were pooled in 2.88 μl β‐mercaptoethanol (Sigma, Poole, Dorset) and 400 μl Solution C (0.322 g guanidine thiocyanate, 377 µl nuclease free water, 23 µl 0.75 M sodium citrate). From each animal approximately 40–60 µg of growth plate tissue was obtained and RNA isolation was performed using proeteinase K digestion followed by phenol:chloroform extraction as previously described (Heinrichs et al., 1994).

### Real‐time quantitative PCR (RT‐qPCR)

RNA content was assessed using a nanodrop spectrophotometer (Thermo Scientific, Chicago, IL) by the absorbance at 260 nm and purity by A260/A280 ratio. Reverse‐transcription was completed as described previously (Farquharson et al., 1999; Houston et al., 1999). RT‐qPCR was performed using the SYBR green (Roche) detection method on a Stratagene Mx3000P real‐time qPCR system (Stratagene, CA) using the following programme: 1 cycle at 95°C for 15 min; 40 cycles at 94°C for 15 sec; 55°C for 30 sec; 72°C for 30 sec; 1 cycle at 95°C for 1 min, 55°C for 30 sec and 95°C for 30 sec. Relative gene expression was calculated using the ΔΔCt method (Livak and Schmittgen, 2001). Each cDNA sample was normalized to housekeeping gene *gapdh* (Primer Design, Southampton, UK) as previously described (Martensson et al 2004). Reactions were performed with gene of interest primers *Igf1*, *Igfbp3, and Igf2* (Invitrogen), as well as *Socs1, Socs2, and Socs3* (Supp. Table 1).

### Immunohistochemistry

Tibiae were dissected from 6‐week‐old male WT (n = 6) and *Socs2^‐/‐^* mice (n = 6) and fixed in 70% ethanol for 48 h at 4°C before decalcification in 10% EDTA (pH 7.4) for approximately 4 weeks at 4°C with regular changes. Tissues were finally dehydrated and embedded in paraffin wax, using standard procedures, and sectioned at 5 µm. For immunohistochemical analysis, sections were dewaxed in xylene and rehydrated before incubation at 37°C for 30 min in 0.1% trypsin (Sigma) for antigen demasking. Endogenous peroxidases were blocked by 0.03% H_2_O_2_ in methanol (Sigma) for 30 min. Antibodies to IGFBP3 and IGF‐2 (both Santa Cruz Biotechnology, Heidelberg, Germany) were diluted to 0.4 μg IgG/ml and 0.5 μg IgG/ml, respectively and incubated for 18 h at 4°C. Control sections were incubated with an equal concentration IgG. A Vectastain Rabbit ABC kit (Vector Laboratories, Peterborough) was then used according to the manufacturer's instructions. The sections were finally dehydrated, counterstained with hematoxylin and mounted in DePeX.

### Conditioned medium analysis

Total IGF‐1 and IGFBP3 levels in conditioned medium from metatarsals cultured ± GH were assessed at days 5, 7, and 12 by ELISA (Quantikine, R&D Systems, Minneapolis, MN). IGF‐2 levels in conditioned medium were assessed at day 12 (Ray Biotech, Inc) according to the manufacturer's instructions. IGF‐1 assays included a step to dissociate the potentially interfering binding proteins from the ligand.

### Statistical analysis

Direct comparison between two sets of data was done by Student's *t*‐test or a suitable non‐parametric test if the data was not normally distributed. Time course experiments were analyzed with a repeated measures 2‐way ANOVA for which suitable post‐tests for multiple comparisons were conducted. Analysis was carried out using SigmaPlot v11.0 (Systat Software Inc, London, UK). Data are presented as mean ± standard error of the mean (SEM). *P* values <0.05 were considered significant.

## Results

### SOCS2 regulation of GH induced growth in metatarsals

**Figure 1:**
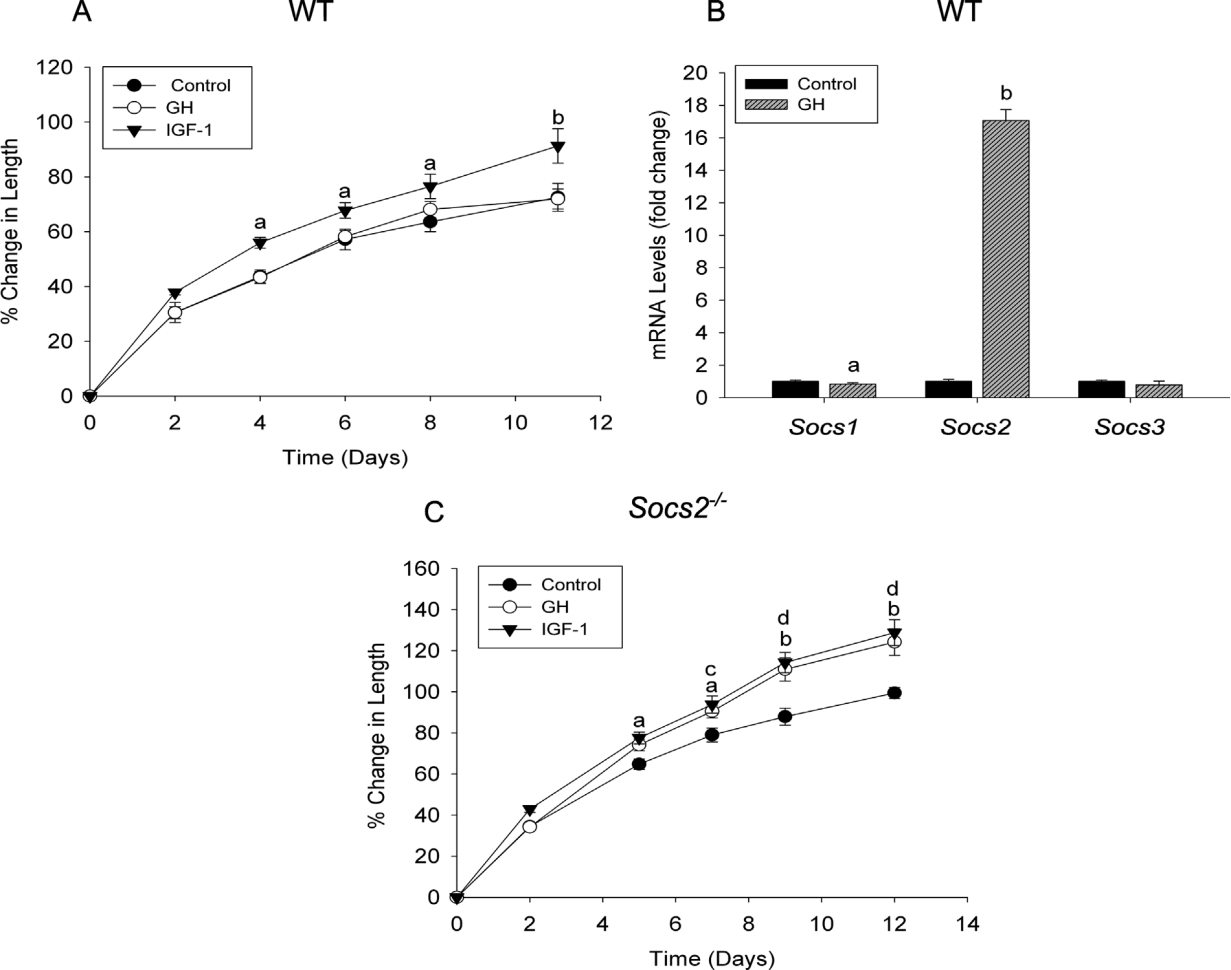
SOCS2 regulation of GH‐induced embryonic metatarsal growth. Graphs showing (A) WT and (C) *Socs2^‐/‐^* E17 metatarsal growth in response to GH or IGF‐1 (100 ng/ml) over a 12 day period. Data are presented as mean ± SEM. Significance denoted by IGF‐1 versus control ^a^ *P *< 0.05, ^b^ *P* < 0.001. GH versus control ^c^ *P* < 0.05, ^d^ *P* < 0.001, (n ≥ 6). (B) Transcript analysis of *Socs1*, *2*, and *3* in WT metatarsals following 7 days GH (100 ng/ml) treatment. Data represented as means ± SEM. Significance from untreated metatarsals denoted by ^a^ *P *< 0.05, ^b^ *P* < 0.01, (n = 3).

WT metatarsals extracted at E17 did not increase their length in response to GH over the 12 day culture period (Fig. 1A). Transcript analysis at day 7, revealed a 17‐fold increase in *Socs2* mRNA expression in the GH‐treated group in comparison to the control group (*P* < 0.01) (Fig. 1B). *Socs1* and *Socs3* mRNA expressions were however not enhanced in response to GH (Fig. 1B). *Socs2^‐/‐^* metatarsals growth significantly increased in response to GH from day 7 to day 12 (*P* < 0.05) (Fig. 1C), similar to previous studies (Pass et al., 2012). IGF‐1 stimulated longitudinal growth in WT and *Socs2^‐/‐^* metatarsals as expected. The level of stimulation in the presence of IGF‐1 was comparable between WT and *Socs2^‐/‐^* metatarsals, indicating that SOCS2 does not regulate IGF‐1 induced growth in this model (Fig. 1A and C). Assessment of WT and *Socs2^‐/‐^* PN3 metatarsals revealed a similar GH response to that noted with the embryonic metatarsal with only the *Socs2^‐/‐^* bones showing enhanced growth in response to GH (*P* < 0.01) (Supp. Fig. 1A and C). Also, *Socs2* but not *Socs1* or *3* mRNA expression was increased in response to 12 days of GH treatment (4‐fold; *P* < 0.01) in WT metatarsals (Supp. Fig. 1B). Taken together, these data indicate that SOCS2 is a major regulator of GH promotion of longitudinal growth in the metatarsal organ culture model.

### Increased growth of *Socs2^‐/‐^* metatarsals in response to GH is not associated with an increase in chondrocyte IGF‐1

**Figure 2:**
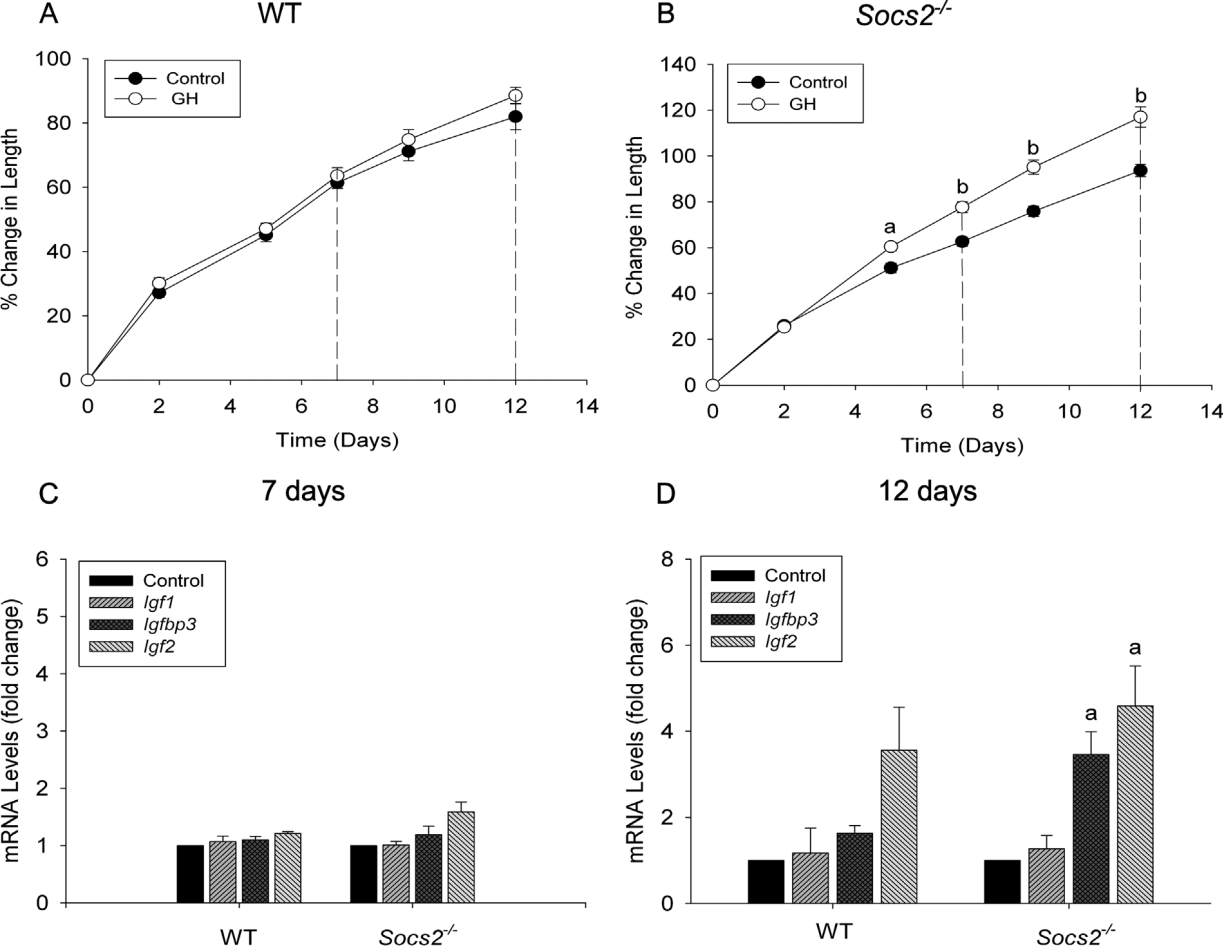
Igf1, Igfbp3, and Igf2 transcript analysis following GH treatment. Graphs showing (A) WT and (B) *Socs2^‐/‐^* E17 metatarsal growth in response to GH (100 ng/ml) over a 12 period. Dotted lines represent points at which *Igf1*, *Igfbp3*, and *Igf2* expression levels were measured. Data are presented as mean ± SEM. Significance from untreated metatarsals denoted by ^a^ *P* < 0.05, ^b^ *P* < 0.01 (n ≥ 6). Transcript analysis of *Igf1*, *Igfbp3*, and *Igf2* in WT and *Socs2^‐/‐^* metatarsals following (C)7‐ and (D)12 days GH (100ng/ml) treatment. Data are presented as mean ± SEM. Significance from untreated metatarsals denoted by ^a^ *P* < 0.05, (n = 3)

We next aimed to determine if the growth promoting effects of GH on *Socs2^‐/‐^* metatarsals were direct or mediated by IGFs. Following 7 days of GH treatment, WT metatarsals showed no increase in longitudinal growth compared to untreated samples, whereas *Socs2^‐/‐^* metatarsal growth increased by 15% (*P* < 0.01) (Fig. 2A and B). Expression levels of *Igf1*, *Igfbp3*, and *Igf2* at this time point were unaltered in response to GH in WT and *Socs2^‐/‐^* metatarsals (Fig. 2C). Protein analysis of day 5 conditioned medium revealed increased IGFBP3 protein levels in GH‐treated *Socs2^‐/‐^* (Control 8.2 ± 1.35, GH 23.5 ± 5.76; *P* < 0.01), but not in corresponding WT conditioned medium (Tables 1 and 2). ELISA analysis of day 7 conditioned medium similarly found no increase in IGF‐1 or IGFBP3 protein levels in GH treated WT metatarsals (Table 1). While IGF‐1 remained at basal level in GH‐stimulated *Socs2^‐/‐^* conditioned medium at day 7, IGFBP3 levels increased significantly (Control 5.2 ± 0.94, GH 22.6 ± 3.34; *P* < 0.01) (Table 2).

**Table 1:** IGF‐1, IGFBP3, and IGF‐2 protein levels in conditioned medium from E17 WT metatarsals following 5, 7, or 12 days GH (100 ng/ml) treatment

Day	Treatment	IGF‐1 (ng/ml)	IGFBP3 (ng/ml)	IGF‐2 (ng/ml)
5	Control	2.4 ± 0.48	9.0 ± 1.43	nd
	GH	2.1 ± 0.23	9.7 ± 1.41	nd
7	Control	6.4 ± 0.86	12.2 ± 1.68	nd
	GH	4.0 ± 0.70	19.7 ± 4.57	nd
12	Control	11.6 ± 1.16	25.0 ± 5.09	7.0 ± 1.39
	GH	9.5 ± 1.06	39.8 ± 3.69^a^	8.4 ± 0.97

Data are presented as mean ± SEM (n ≥ 5). Significance from day matched control samples denoted by ^a^ *P* < 0.05. nd = no data.

**Table 2:** IGF‐1, IGFBP3, and IGF‐2 protein levels in conditioned medium from E17 *Socs2^‐/‐^* metatarsals following 5, 7, or 12 days GH (100 ng/ml) treatment

Day	Treatment	IGF‐1 (ng/ml)	IGFBP3 (ng/ml)	IGF‐2 (ng/ml)
5	Control	2.7 ± 0.59	8.2 ± 1.35	nd
	GH	2.6 ± 0.26	23.5 ± 5.76^b^	nd
7	Control	3.3 ± 0.39	5.2 ± 0.94	nd
	GH	2.8 ± 0.28	22.6 ± 3.34^b^	nd
12	Control	9.9 ± 0.90	22.2 ± 3.51	1.7 ± 0.34
	GH	7.0 ± 0.39	49.6 ± 1.89^c^	17.1 ± 1.72^c^

Data are presented as mean ± SEM (n ≥ 5). Significance from control day matched control samples denoted by ^b^ *P* < 0.01, ^c^ *P* < 0.001.

At day 12, *Socs2^‐/‐^* metatarsal growth increased by 23% (*P* < 0.01) in response to GH, while WT metatarsal growth remained at control level (Fig. 2A and B). Similar to the day 7 data; expression levels of *Igf1*, *Igfbp3*, and *Igf2* were unaltered in response to GH in WT metatarsal (Fig. 2D). Similarly, *Igf1* transcript levels remained at basal level in GH‐treated *Socs2^‐/‐^* metatarsals, whereas *Igfbp3* and *Igf2* transcript levels significantly increased by 3.5‐ (*P* < 0.05) and 4.6‐ fold (*P* < 0.05) respectively (Fig. 2D). A similar trend was observed when analyzing protein levels of conditioned medium (Tables 1 and 2). GH failed to have a stimulatory effect on IGF‐1 levels in WT or *Socs2^‐/‐^* conditioned medium. However, GH treatment did result in a 59% (*P* < 0.01) and 123% (*P* < 0.001) increase in IGFBP3 levels in WT and *Socs2^‐/‐^* conditioned medium, respectively. IGF‐2 protein levels were also increased in *Socs2^‐/‐^* conditioned medium in response to GH (*P* < 0.001) (Table 2). No increase in IGF‐2 levels was observed in corresponding WT medium (Table 1).

Experiments carried out on PN3 metatarsals produced comparable results to those observed in E17 metatarsals in terms of IGF‐1 production. Despite there being an increase in growth of *Socs2^‐/‐^* metatarsals in response to GH, IGF‐1 levels in conditioned medium at day 7 and 12 were not preferentially increased in *Socs2^‐/‐^* metatarsals (Supp. Tables 2 and 3). GH stimulated IGFBP3 protein levels in WT‐conditioned medium, an increase that appeared to be enhanced in the absence of SOCS2 at day 12 (Supp. Tables 2 and 3). A comparable increase in IGF‐2 levels in conditioned medium following GH treatment was observed in WT and *Socs2^‐/‐^* metatarsals (Supp. Tables 2 and 3).

These data reveal that increased longitudinal growth in response to GH observed in the *Socs2^‐/‐^* metatarsal model is not mediated by increased IGF‐1 expression. They also highlight the possible importance of IGFBP3 and IGF‐2 in this model as secondary messengers to GH in promoting longitudinal bone growth in *Socs2^‐/‐^* metatarsals.

### Altered IGF‐2 and IGFBP3 levels in the growth plates of *Socs2^‐/‐^* mice

**Figure 3:**
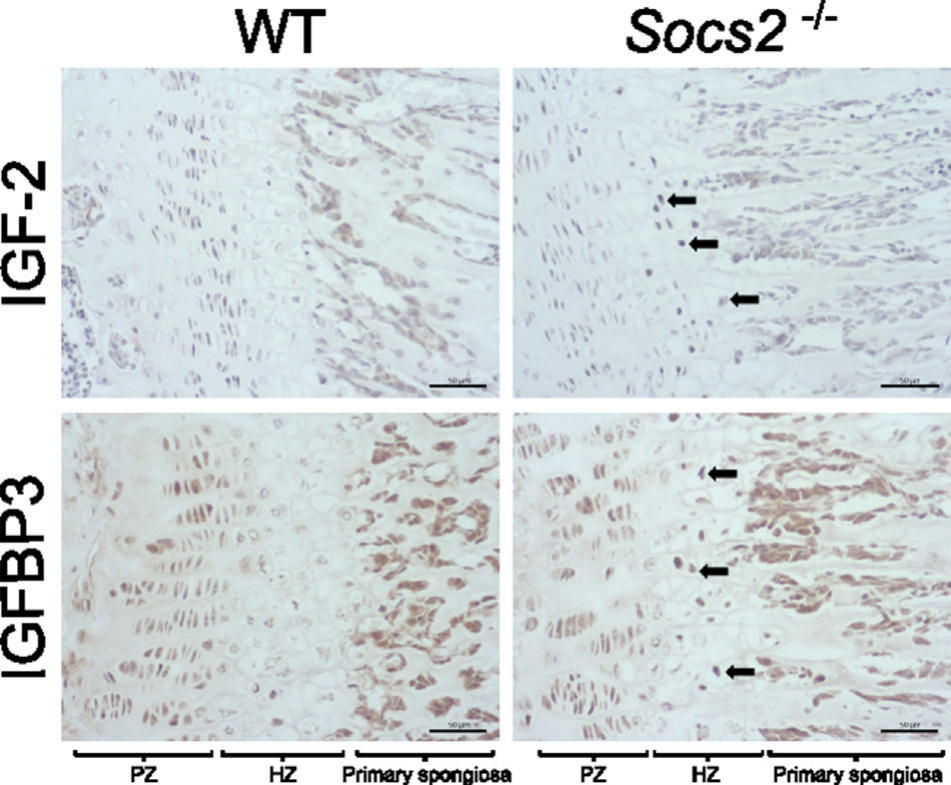
Immunohistochemical localization of IGF‐2 and IGFBP3 to the proliferating and hypertrophic chondrocytes of WT and Socs2^‐/‐^ growth plates. In contrast to WT growth plates, hypertrophic chondrocytes within the Socs2^‐/‐^ growth plate expressed high levels of both IGF‐2 and IGFBP3 (arrows). Scale bars = 50μm

Examination of growth plates from WT mice indicated that both IGF‐2 and IGFBP3 protein were preferentially localized to the proliferating zone of the growth plate with little or no staining in the hypertrophic chondrocytes. The staining in the proliferating chondrocytes of the *Socs2^‐/‐^* growth plates of both IGF‐2 and IGFBP3 appeared similar to that observed in similarly stained WT growth plates. In contrast, growth plates from *Socs2^‐/‐^* mice contained a greater number of hypertrophic chondrocytes that stained positively for both IGF‐2 and IGFBP3 (Fig. 3). All control sections showed no staining (data not shown).

### No additive effects of GH and IGF‐1 action in the promotion of metatarsal linear growth

**Figure 4:**
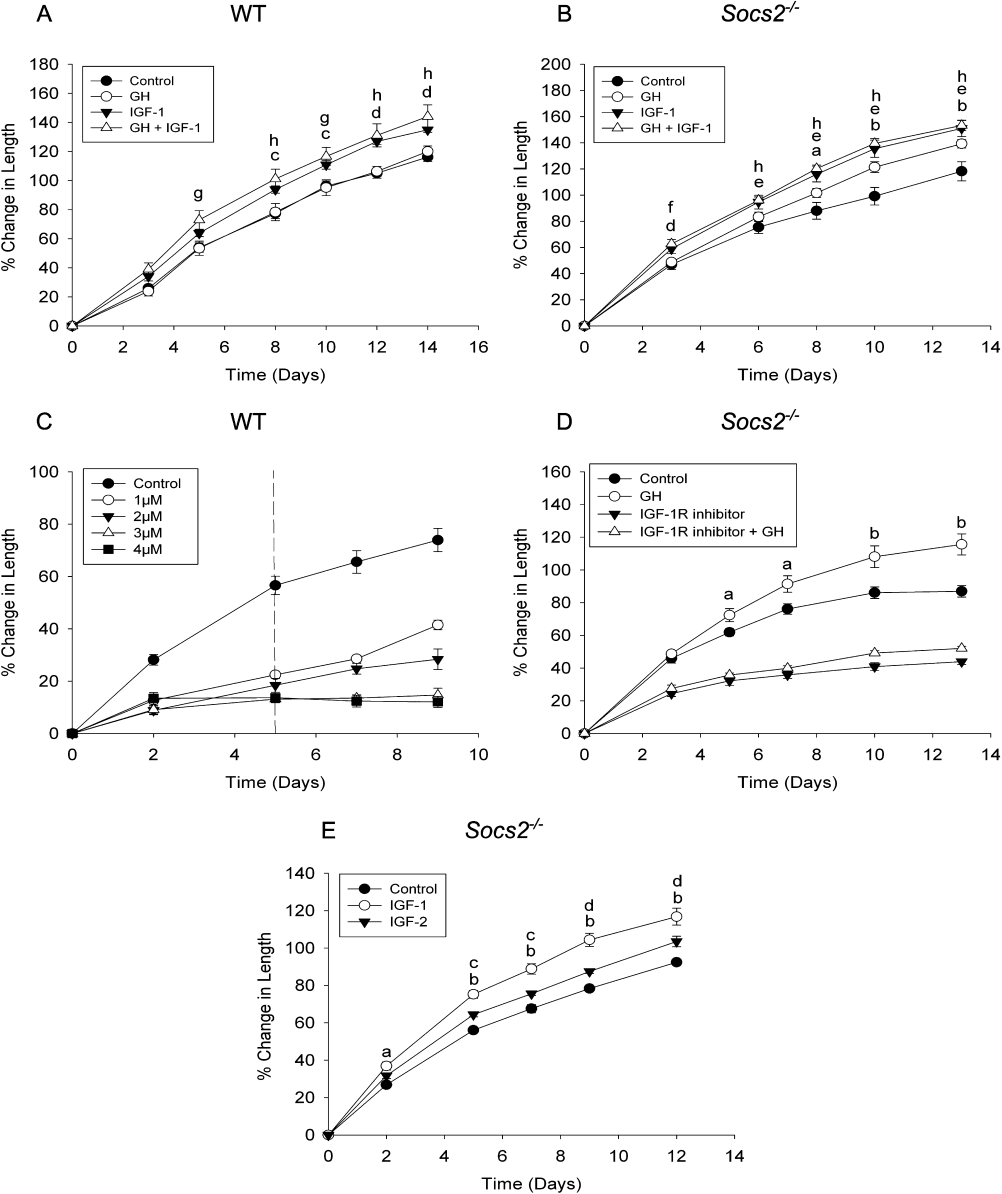
Effect of IGF‐1R inhibitor, IGF‐2, and combined IGF‐I + GH on metatarsal growth. Graphs showing (A) WT and (B) *Socs2^‐/‐^* E17 metatarsal growth in response to GH or IGF‐1 (100ng/ml) or GH + IGF‐1 over a 13–14 day period. Data are presented as mean ± SEM. Significance denoted by GH versus control ^a^ *P* < 0.05, ^b^ *P* < 0.001. IGF‐1 versus control ^c^ *P* < 0.05, ^d^ *P* < 0.01, ^e^ *P* < 0.001, GH+IGF‐1 versus control ^f^ *P* < 0.05, ^g^ *P* < 0.01, ^h^ *P* < 0.001, (n ≥ 6). (C) Graph showing WT E17 metatarsal growth in response to NVP‐AEW481 (1–4 μM) for 5 days with 4 days recovery. Data are presented as mean ± SEM. (n ≥ 6). (D) Graph showing *Socs2^‐/‐^* E17 metatarsal growth in response to GH (100 ng/ml), NVP‐AEW481 (1 μM) or GH + NVP‐AEW481. Data are presented as mean ± SEM. Significance of GH versus control denoted by ^a^ *P* < 0.05, ^b^ *P *< 0.01, (n ≥ 6). (E) Graph showing *Socs2^‐/‐^* E17 metatarsal growth in response to IGF‐1 or IGF‐2 (100 ng/ml). Data are presented as mean ± SEM. Significance denoted by IGF‐1 versus control ^a^ *P* < 0.01, ^b^ *P* < 0.001. IGF‐2 versus control ^c^ *P* < 0.05 ^d^ *P* < 0.01, (n ≥ 6).

Having shown that both GH and IGF‐1 stimulated longitudinal growth in *Socs2^‐/‐^* metatarsals, and that GH stimulation was independent of increased IGF‐1 levels, we next aimed to determine if the actions of GH and IGF‐1 on longitudinal growth were additive. Dual treatment with GH and IGF‐1 in WT metatarsals significantly increased growth in comparison to control samples (*P* < 0.001) (Fig. 4A). This level of increase did not differ from that observed with IGF‐1 treatment alone. In *Socs2^‐/‐^* metatarsals, growth was significantly increased in response to both IGF‐1 and GH treatment in comparison to control samples (*P* < 0.01, *P* < 0.05 respectively; Fig. 4B). There was no additional increase in metatarsal growth upon dual treatment (Fig. 4B).

### The IGF‐1R is critical for GH‐induced growth

The cellular responses of IGF‐1 and IGF‐2 are both mediated through the IGF‐1R. To investigate the importance of this receptor in mediating the effects of GH on longitudinal bone growth, we assessed the growth of *Socs2^‐/‐^* metatarsals in the presence of GH and an IGF‐1R inhibitor (NVP‐AEW541). Initially, WT metatarsals were treated with varying concentrations of NVP‐AEW541 for 5 days before the inhibitor was removed from the medium (Fig. 4C). All concentrations of NVP‐AEW541 (1–4 μM) inhibited metatarsal growth confirming that endogenous IGF signaling is important for metatarsal growth. One micrometer of NVP‐AEW541 was selected for future experiments as on the removal of the inhibitor, metatarsals showed increased growth suggesting that the inhibitor at this concentration (*cf* 3 and 4 μM) had no lasting toxic effects on growth potential. *Socs2^‐/‐^* metatarsals treated with the NVP‐AEW541 showed significantly decreased growth compared to control samples (*P* < 0.05, Fig. 4D). GH treatment was unable to stimulate growth in the presence of the IGF‐1R inhibitor, suggesting that the effects of GH on longitudinal bone growth are mediated through the IGF‐1R.

### IGF‐2 stimulation of embryonic *Socs2^‐/‐^* metatarsals

As GH‐induced growth in *Socs2^‐/‐^* metatarsals was associated with an increase in IGF‐2 levels (Table 2), we next determined if IGF‐2 could stimulate longitudinal growth. IGF‐2 treatment significantly increased longitudinal growth of *Socs2^‐/‐^* metatarsals from day 5 to 12 (*P* < 0.05; Fig. 4E). This enhanced growth by IGF‐2 was however, significantly less than that stimulated by equimolar IGF‐1, indicating that IGF‐1 is the more potent of the two IGF ligands in promoting linear bone growth (Fig. 4E). Similar results were observed in WT metatarsals (data not shown).

### Igf1 mRNA levels in the Socs2^‐/‐^ growth plate

**Figure 5:**
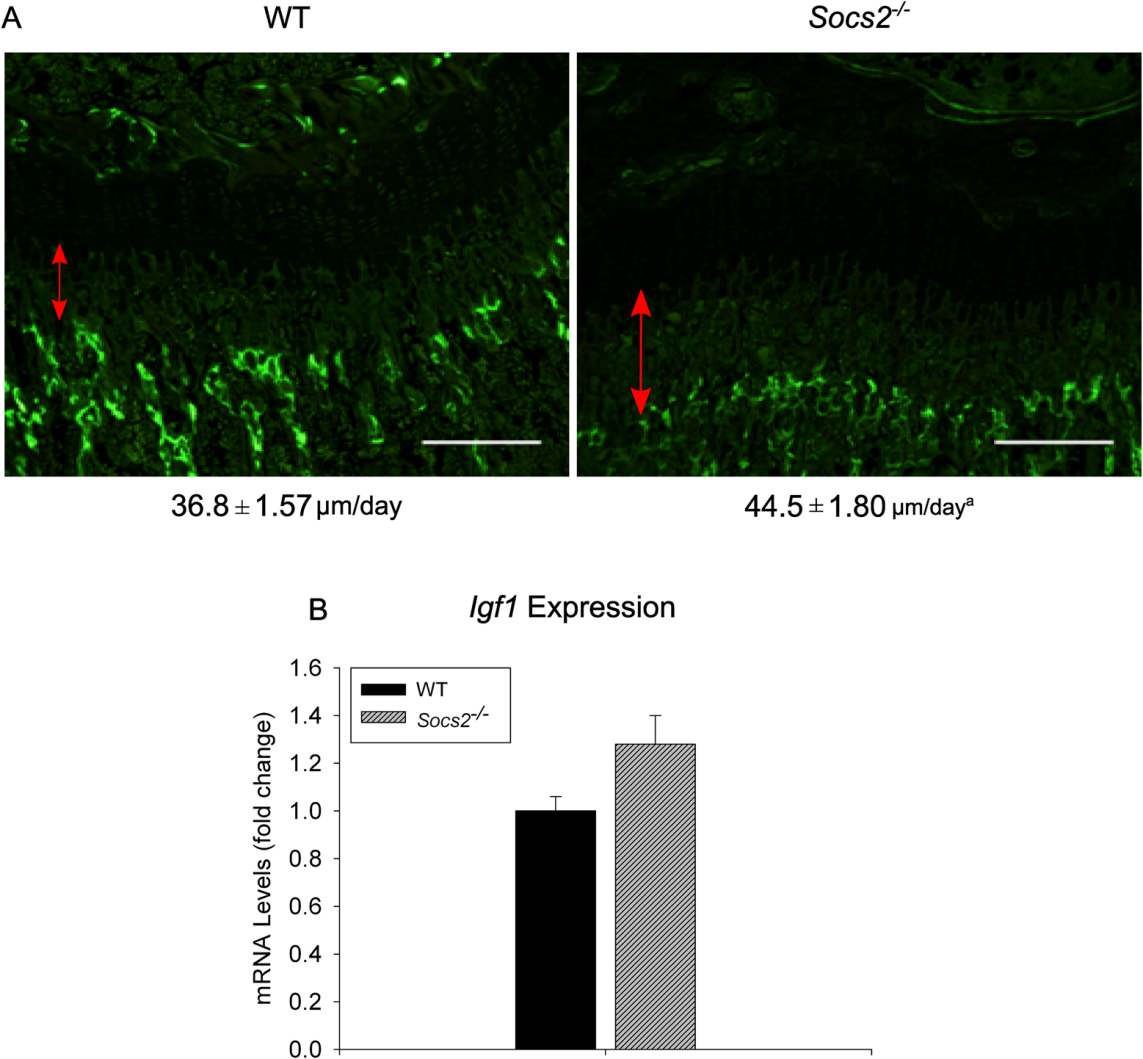
Normal IGF‐1 mRNA expression in Socs2^‐/‐^ growth plate. (A) Analysis of longitudinal bone growth rate by dynamic histomorphometry of 6‐week‐old male WT and *Socs2^‐/‐^* mice. The red lines indicate the distance between the original growth plate mineralization front at the proximal end of the tibia and the fluorescing mineralization front. The numbers under the images indicate longitudinal bone formation rate (μm/day). Scale bars = 100 μm. Data presented as mean ± SEM. Significance denoted by ^a^ *P* < 0.05, (n ≥ 6). (B) Transcript analysis of *Igf1* expression in WT and *Socs2^‐/‐^* growth plates, micro‐dissected from 7‐week‐old male mice. Data are presented as fold change relative to WT expression as mean ± SEM, (n ≥ 3).

Calcein labeling of 6‐week‐old *Socs2* ^‐/‐^ mice revealed that tibial growth over a 2 day period of *Socs2^‐/‐^* mice (44.5 ± 1.8μm) was increased in comparison to WT mice (36.8 ± 1.57 μm) (21%; p<0.05) (Fig. 5A). *Igf1* levels were also assessed in the growth plates of WT and *Socs2^‐/‐^* mice and found not to be significantly altered from expression levels of WT mice. Therefore, despite increased bone growth rates in *Socs2^‐/‐^* mice there was no elevation in growth plate *Igf1* transcript levels (Fig. 5B).

## Discussion

It is widely understood that GH action on growth plate chondrocytes is an important regulator of linear growth (Ahmed and Farquharson, 2010). The intimate relationship between GH and IGF‐1 does however make it difficult to attribute specific actions to GH, or those resulting through IGF‐1. Tibial growth is reduced in both *ghr* and *Igf1* null mice. This reduction in growth becomes more severe in the double *ghr/igf1* mutants, suggesting that GH and IGF‐1 have independent functions (Lupu et al., 2001). In agreement with this, mice deficient of GH show a much higher reduction in longitudinal bone growth than *Igf1* null mice (Sims et al., 2000; Mohan et al., 2003; Wang et al., 2004). The present study demonstrates that despite no elevation in IGF‐1 gene and protein expression levels in *Socs2^‐/‐^* metatarsals following GH treatment, GH enhances linear bone growth. This indicates that the growth promoting effects of GH are not accompanied by a rise in IGF‐1 expression by chondrocytes or cells of the perichondrium, which previous studies have indicated is a major source of *Igf1* in the growing bone (Parker et al., 2007). This was supported by the observation of normal *Igf1* expression levels in the growth plates of *Socs2^‐/‐^* tibia which are characterized by wider growth plates and longer bones (MacRae et al., 2009; Pass et al., 2012).

In addition to their normal GH and IGF‐1 serum concentrations, *Socs2* ^‐/‐^ mice also have normal carbohydrate metabolism compared with ALSKO, BP3KO, LID, and IGF‐1 null mice (Rico‐Bautista et al., 2005; Yakar et al., 2009). Thus, the *Socs2* ^‐/‐^ mouse may represent an important model to study local autocrine/paracrine pathways that promote linear bone growth without leading to insulin resistance and altered carbohydrate metabolism.

Several studies have used the fetal mouse metatarsal culture methods to investigate the anabolic effects of IGF‐1 on linear bone growth. Increased growth of metatarsals in response to IGF‐1 is coupled with an increase in proliferation and an increase in hypertrophic zone size (Mushtaq et al., 2004; MacRae et al., 2006, 2007; Chagin et al., 2010). The effects of GH on metatarsal growth are however less clear, with GH being shown to either stimulate or have no effect on growth (Scheven and Hamilton, 1991; Mushtaq et al., 2004; Pass et al., 2012). In chondrocytes, GH is able to stimulate SOCS2 expression (Pass et al., 2012) and we have now further shown that SOCS2 is a negative regulator of GH's capability to promote longitudinal bone growth in the metatarsal organ culture model. This observation is in agreement with previous reports where mice missing the *Socs2* gene have increased linear bone growth due to increased signaling through the GHR (Metcalf et al., 2000; Greenhalgh et al., 2005; MacRae et al., 2009). Although, it is well recognized that GH signaling is also modulated by CIS and SOCS1 and 3 we have reported previously that GH does not stimulate chondrocyte SOCS 1 or 3 protein levels (Pass et al., 2009, 2012). Furthermore, *Socs1* ^‐/‐^ and *Socs3* ^‐/‐^ mice are perinatal and embryonic lethal, respectively and little data exists on their ability to regulate bone growth (Greenhalgh and Alexander, 2004). While further studies are required to investigate GH ability to stimulate chondrocyte CIS expression it is of interest to note that *Cis* ^‐/‐^ mice exhibit no obvious growth phenotype (Greenhalgh and Alexander, 2004).

It is widely established that the prenatal growth promoting effects of IGF‐1 are GH independent, and that GH has little or no role in promoting growth in embryonic development (Waters and Kaye, 2002). Therefore, these key studies were also completed using postnatal metatarsals, which may be more sensitive to the growth promoting actions of GH. No evidence for this was however observed in our experiments and older postnatal bones may have to be studied to see a GH response. However, this was not possible as our pilot studies (data not shown) indicated that 3‐day‐old postnatal metatarsals was the oldest age we could study to get measurable growth. Even at postnatal day 3, bone growth was much less than embryonic metatarsals and this is in accord with previous observations (Macrae et al. and 2006; Chagin et al., 2010). It is therefore tantalizing to speculate that variable SOCS2 expression in normal animals may “fine tune” the chondrocytes ability to respond to GH. Such a regulatory mechanism may permit postnatal bones at a specific developmental age to respond to GH and further studies are required to investigate this.

*Socs2^‐/‐^* mice have increased linear bone growth consistent with increased signaling through the GHR (Metcalf et al., 2000; Greenhalgh et al., 2005; Lorentzon et al., 2005; MacRae et al., 2009; Pass et al., 2012). As *Socs2^‐/‐^* mice are devoid of higher circulating IGF‐1 levels, it is likely that the increased linear bone growth and structural alterations within *Socs2^‐/‐^* growth plate are a direct consequence of GH acting directly on growth plate cartilage (Lorentzon et al., 2005; MacRae et al., 2009). The broad distribution of both the GHR and IGF‐1R within the growth plate suggests GH/IGF‐1 may play a role in chondrocyte proliferation, differentiation, and hypertrophy (Lupu et al., 2001; Gevers et al., 2002b; Wang et al., 2011). The germinal zone of the growth plates are significantly enlarged in *Igf1* null mice, supporting the idea that GH stimulates longitudinal bone growth independent of IGF‐1 (by stimulating the differentiation of growth plate precursor cells), and via an IGF‐1 dependent mechanism (by inducing *Igf1* expression in differentiating chondrocytes which then in‐turn stimulate clonal expansion) (Isaksson et al., 1987; Wang et al., 2004). Although IGF‐1 is expressed in all maturational growth plate zones, it is understood to act mainly on the hypertrophic zone. *Igf1^‐/‐^* mice have attenuation of chondrocyte hypertrophy with no changes in proliferation (Wang et al., 1999). Addition of IGF‐1 to metatarsals leads to an increase in hypertrophic zone lengths as well as increased size of individual chondrocytes (Mushtaq et al., 2004). These mechanistic observations are important as a large part of growth of cultured metatarsals is due to hypertrophic differentiation but it is recognized these observation may not translate directly to the in vivo situation where matrix production, proliferation and hypertrophy all contribute to linear bone growth (Wilsman et al., 1996).

The GH signaling mechanisms responsible for increasing linear bone growth have been recently clarified in studies where GH was found to stimulate the phosphorylation of STATs 1, 3, and 5 in chondrocyte cultures (Pass et al., 2012). The activation of chondrocyte STAT signaling was both increased and prolonged in chondrocytes from *Socs2^‐/‐^* mice in comparison to cells from WT mice (Pass et al., 2012). This explains the observed GH stimulation of linear growth of *Socs2^‐/‐^* embryonic metatarsals and the proliferation of chondrocytes within (Pass et al., 2012).

Both IGF‐1 and IGF‐2 are important regulators of skeletal growth. While IGF‐1 is important for both fetal and postnatal growth, IGF‐2 is thought to function only in the former. IGF‐2 is highly expressed in a variety of tissues in the embryo, and is dramatically down‐regulated shortly after birth (Lui et al., 2008). Disruption of the *Igf2* gene results in progeny that are 60% smaller than WT littermates, however postnatal growth is comparable to WT (Dechiara et al., 1990). Although it is widely understood that IGF‐2 actions are independent of GH regulation, GH has been shown to regulate IGF2 transcription in the human liver (von Horn et al., 2002). In this present study, both during fetal and early postnatal linear growth, IGF‐2 levels are regulated by GH. It is conceivable that the increase in linear growth observed in *Socs2^‐/‐^* metatarsals are a result of elevated IGF‐2 levels. IGF‐2 can promote the expansion of fetal hypertrophic chondrocytes in cultured limb explants, possibly through activation of the PI3K and TGF‐ß pathways and downstream elevation of transcription factors (Hamamura et al., 2008; Chen et al., 2010). Both IGF‐1 and IGF‐2 bind to the IGF‐1R, and the cellular responses of both ligands are mediated through this receptor. As inhibition of this receptor abrogates the growth promoting effects of GH, it stands to reason that IGF's are responsible for mediating the increase in linear growth. As there is no increase in the expression of IGF‐1 in response to GH, it is possible that IGF‐2 and not IGF‐1 is responsible for the GH promoted linear growth of the *Socs2^‐/‐^* metatarsals. In agreement with this, IGF‐2 was able to stimulate longitudinal bone growth in metatarsals from WT and *Socs2^‐/‐^* mice.

Confirmation of increased expression of IGF‐2 and IGFBP3 by the hypertrophic chondrocytes of growth plates in response to unrestricted GH signaling was revealed by immunohistochemical analysis of *Socs2^‐/‐^* growth plates. The presence of IGF‐2 and IGFBP3 in hypertrophic chondrocytes is consistent with GHR expression in these cells of the growth plate and our previous observation of increased numbers of phosphorylated STAT‐5–positive hypertrophic chondrocytes of *Socs2^‐/‐^* growth plates (Gevers et al., 2002b; Pass et al., 2012). The staining in the proliferating chondrocytes of the *Socs2^‐/‐^* growth plates appeared similar to that observed in the WT growth plates however it was not possible, because of the nonquantitative nature of immunohistochemistry, to compare levels of IGF‐2 and IGFBP3 in the proliferating chondrocytes. These data suggest that SOCS2 can influence hypertrophic chondrocyte IGF‐2 and IGFBP3 availability and thereby modulate longitudinal bone growth.

The growth rate of tibia from *ghr/Igf1* null mice are almost identical to the sum of growth deficit observed in the single *ghr* and *igf1r* mutants suggesting that GH and IGF‐1 have independent roles in promoting linear growth (Lupu et al., 2001). Local administration of GH+IGF‐1 however failed to have an additive effect in hypophysectomised animals (Isgaard et al., 1986). In agreement with this, we show that in *Socs2^‐/‐^* metatarsals, dual treatment with GH and IGF‐1 does not promote longitudinal growth beyond individual stimulation. This suggests that GH is promoting linear growth through a common pathway with IGF‐1. It is however plausible that the increase in IGF‐2 levels observed in *Socs2^‐/‐^* metatarsals observed in this study are competing with the exogenous IGF‐1. As IGF‐1 exhibits a higher binding affinity to the IGF‐1R than IGF‐2 it is likely that the growth promoting effects of IGF‐2 would be masked (Danielsen et al., 1990; Germainlee et al., 1992; Oh et al., 1993).

In the present study GH stimulation also causes significant increase in IGFBP3 levels although, like IGF‐1, the source of this IGFBP3 maybe both chondrocytes and/or the surrounding perichondrium (Parker et al., 2007). The increase is more apparent in *Socs2^‐/‐^* metatarsals, where growth is promoted. IGFBP3 is produced in a number of tissues and is regulated mainly by GH, but also to some degree by IGF‐1 (Kiepe et al., 2005). It is the principal carrier of IGFs in serum, where it functions to control tissue IGF concentrations and reduce bioavailability (Clemmons, 1998). Depending on incubation time and dose, it can inhibit or potentiate the actions of IGFs (Demellow and Baxter, 1988; Stewart et al., 1993; Bagi et al., 1994). IGFBP3 may have pro‐apoptotic functions on chondrocytes, independent of IGFs, which is likely mediated through its own cell surface bound receptor (Oh et al., 1993; Spagnoli et al., 2002). The complex relationship between IGF's and IGFBP3 make it difficult to deduce what its role may be in this system. As there appears to be a correlation between increased IGFBP3 and increased growth in response to GH, it is unlikely that the effects of IGFBP3 are inhibitory in this model.

In conclusion, using the murine metatarsal model, this study underscores the critical role of SOCS2 in controlling GH anabolic effects on linear bone growth. This study also provides compelling evidence to support the notion that in the absence of SOCS2, GH can regulate linear growth via local mechanisms that are not accompanied by a rise in local or circulating IGF‐1.

## Supporting information

Additional supporting information may be found in the online version of this article at the publisher's web‐site.

**Supporting Information Figure S1**: SOCS2 regulation of GH induced postnatal metatarsal growth. Graphs showing (A) WT and (C) Socs2^‐/‐^ postnatal (PN) 3 metatarsal growth in response to GH (100 ng/ml) over a 12 day period. Dotted lines indicate points at which conditioned medium analysed. Data are presented as mean ± SEM. Significance GH versus control denoted by ^a^ *P* < 0.05, ^b^ *P* < 0.01, (n ≥ 6). (B) Transcript analysis of Socs1, 2, and 3 in WT metatarsals following 12 days GH (100 ng/ml) treatment. Data represented as means ± SEM. Significance from untreated metatarsals denoted by ^b^ *P* < 0.01, (n = 3).

**Supporting Information Table S1**: Primers used for genotyping and PCR analysis.
**Supporting Information Table S2**: IGF‐1, IGFBP3, and IGF‐2 protein levels in conditioned medium from PN3 WT metatarsals following 7, or 12 days GH (100 ng/ml) treatment.
**Supporting Information Table S3**.
