# Supplementary figures and images for: Increased linear bone growth by GH in the absence of SOCS2 is independent of IGF‐1
Source: J Cell Physiol. 2015 Jul 27;230(11):2796–806. doi: 10.1002/jcp.25006 (PMC4949688; doi:10.1002/jcp.25006)

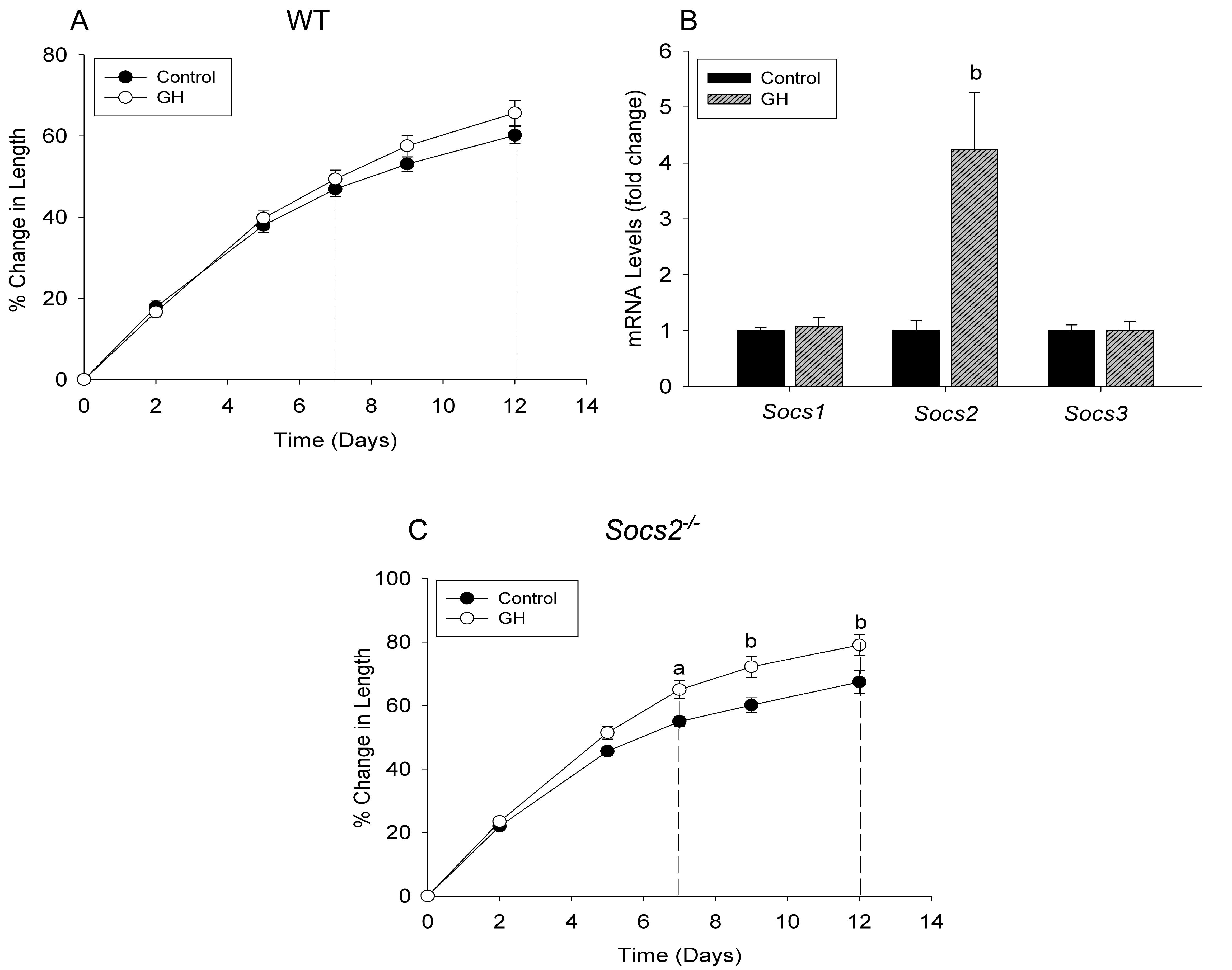

Supplement: Supplementary file 1 — Supporting Information Figure S1: SOCS2 regulation of GH induced postnatal metatarsal growth. Graphs showing (A) WT and (C) Socs2‐/‐ postnatal (PN) 3 metatarsal growth in response to GH (100 ng/ml) over a 12 day period. Dotted lines indicate points at which conditioned medium analysed. Data are presented as mean ± SEM. Significance GH versus control denoted by a P < 0.05, b P < 0.01, (n ≥ 6). (B) Transcript analysis of Socs1, 2, and 3 in WT metatarsals following 12 days GH (100 ng/ml) treatment. Data represented as means ± SEM. Significance from untreated metatarsals denoted by b P < 0.01, (n = 3). [file JCP-230-2796-s001.tif]
