# Supplementary material for: Increased linear bone growth by GH in the absence of SOCS2 is independent of IGF‐1
Source: J Cell Physiol. 2015 Jul 27;230(11):2796–806. doi: 10.1002/jcp.25006 (PMC4949688; doi:10.1002/jcp.25006)
Supplement: Supplementary file 2 — Supporting Information Table S1: Primers used for genotyping and PCR analysis. Supporting Information Table S2: IGF‐1, IGFBP3, and IGF‐2 protein levels in conditioned medium from PN3 WT metatarsals following 7, or 12 days GH (100 ng/ml) treatment. Supporting Information Table S3. [file JCP-230-2796-s002.docx]

**Supp. Table 1.** Primers used for genotyping and PCR analysis

|  | **Forward (5'-3')** | **Reverse (5'-3')** |
| --- | --- | --- |
| Genotyping |  |  |
| SOCS2 | TGTTTGACTGAGCTCGCGC | CAACTTTAGTGTCTTGGATCT |
| Neo | ACCCTGCACACTCTCGTTTTG | CCTCGACTAAACACATGTAAAGC |
| PCR Analysis | |  |
| *Socs1* | TCCGATTACCGGCGCATCACG | CTCCAGCAGCTCGAAAAGGCA |
| *Socs2* | TGGCTGCTCAAGATCAAATG | TGTCCTCCTGGAAATGGAAG |
| *Socs3* | GAGTACCCCCAAGAGAGCTTACTA | CTCCTTAAAGTGGAGCATCATACTG |

| **Day** | **Treatment** | | **IGF-1 (ng/ml)** | | |  | **IGFBP3 (ng/ml)** | | |  | **IGF-2 (ng/ml)** | | |
| --- | --- | --- | --- | --- | --- | --- | --- | --- | --- | --- | --- | --- | --- |
| **7** | Control |  | 3.4 | ± | 0.24 |  | 5.4 | ± | 2.16 |  | 3.0 | ± | 0.37 |
|  | GH |  | 3.5 | ± | 0.71 |  | 16.5 | ± | 4.68^a^ |  | 10.9 | ± | 3.17^b^ |
| **12** | Control |  | 7.3 | ± | 0.91 |  | 14.5 | ± | 4.86 |  | 5.6 | ± | 0.80 |
|  | GH |  | 10.9 | ± | 1.20^a^ |  | 39.4 | ± | 5.00^b^ |  | 11.0 | ± | 1.61^a^ |

**Supp. Table 2.** IGF-1, IGFBP3 and IGF-2 protein levels in conditioned medium from PN3 WT metatarsals following 7, or 12 days GH (100ng/ml) treatment.

Data are presented as mean ± SEM (n≥5). Significance from day matched control samples denoted by ^a^ p<0.05, ^b^ p<0.01.

**Supp. Table 3.** IGF-1, IGFBP3 and IGF-2 protein levels in conditioned medium from PN3 *Socs2^-/-^* metatarsals following 7, or 12 days GH (100ng/ml) treatment.

| **Day** | **Treatment** | **IGF-1 (ng/ml)** | | |  | **IGFBP3 (ng/ml)** | | |  | **IGF-2 (ng/ml)** | | |
| --- | --- | --- | --- | --- | --- | --- | --- | --- | --- | --- | --- | --- |
| **7** | Control | 4.6 | ± | 0.54 |  | 21.1 | ± | 7.2 |  | 3.13 | ± | 0.87 |
|  | GH | 4.4 | ± | 0.67 |  | 40.0 | ± | 10.0 |  | 10.67 | ± | 1.49^c^ |
| **12** | Control | 10.5 | ± | 2.08 |  | 35.9 | ± | 18.4 |  | 7.04 | ± | 1.66 |
|  | GH | 13.6 | ± | 1.70 |  | 130.4 | ± | 28.2^a^ |  | 12.86 | ± | 1.93^a^ |

Data are presented as mean ± SEM (n≥5). Significance from day matched control samples denoted by ^a^ p<0.05, ^c^ p<0.001.
